# Supplementary material for: Differential mitochondrial protein interaction profile between human translocator protein and its A147T polymorphism variant
Source: PLoS One. 2022 May 6;17(5):e0254296. doi: 10.1371/journal.pone.0254296 (PMC9075623; doi:10.1371/journal.pone.0254296)
Supplement: S1 Fig — A-B) 14-3-3 η protein (YWHAH) and 14-3-3 β (YWHAB) were expressed in U87MG cells together with V5-tagged human TSPOWT or TSPOA147T. A) Co-IP of YWHAH and hTSPOWT or hTSPOA147T with V5 antibody and detected by immunoblotting for myc. YWHAH was not co-purified with V5. B) Co-IP of YWHAB and hTSPOWT or hTSPOA147T with V5 antibody and detected by immunoblotting for myc. YWHAB was not co-purified with V5. (PDF) [file pone.0254296.s001.pdf]

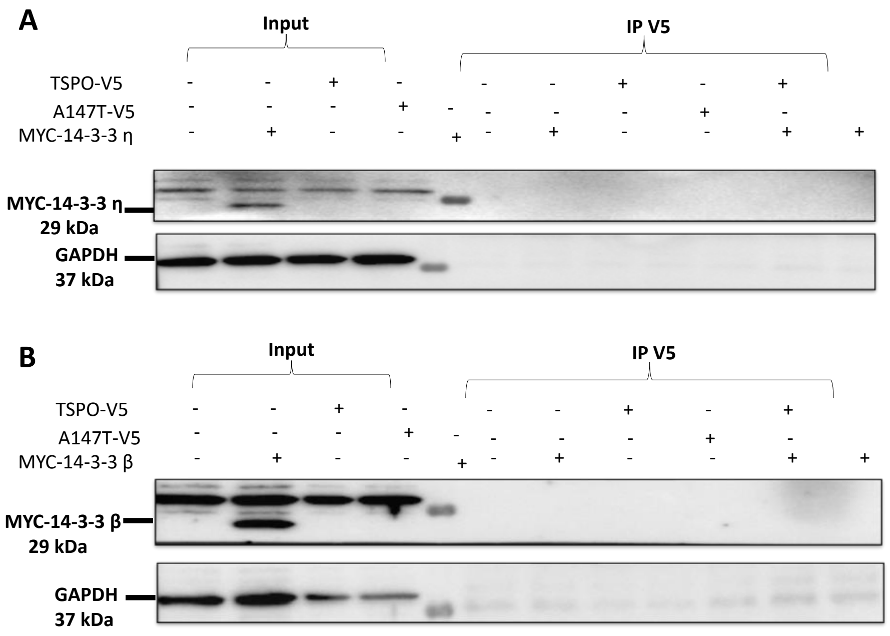

Fig S1. Validated non-interactors of hTSPO^{WT} and hTSPO^{A147T}. A-B) 14-3-3 η protein (YWHAH) and 14-3-3 β (YWHAB) were expressed in U87MG cells together with V5-tagged human TSPO^{WT} or TSPO^{A147T}. A) Co-IP of YWHAH and hTSPO^{WT} or hTSPO^{A147T} with V5 antibody and detected by immunoblotting for myc. YWHAH was not co-purified with V5. B) Co-IP of YWHAB and hTSPO^{WT} or hTSPO^{A147T} with V5 antibody and detected by immunoblotting for myc. YWHAB was not co-purified with V5.
