# Supplementary material for: Differential mitochondrial protein interaction profile between human translocator protein and its A147T polymorphism variant
Source: PLoS One. 2022 May 6;17(5):e0254296. doi: 10.1371/journal.pone.0254296 (PMC9075623; doi:10.1371/journal.pone.0254296)
Supplement: Attachment — Submitted filename: PtP letter revision.pdf [file pone.0254296.s003.pdf]

Point-to-point response to reviewer comments

We thank both reviewers for their comments that have been addressed as indicated below. We hope you find the revised version of the manuscript ready for publication in PLOS ONE.

Reviewer #1: The manuscript of the paper entitled "Differential mitochondrial protein interaction profile between human translocator protein and its A147T polymorphism variant' is presented in a proper manner. However, author should consider following corrections before resubmission:
1.Page 10, L16 replace 'cOmplete with 'complete'
2.Page 10, L17; Page 10, L 22; Page 11, L 6, replace '))' with ')'
3.Page 11, L 13 replace 'inhibitor .' with 'inhibitor.'
4.Maintain consistency in units such as 50 mM instead of 50mM, 4oC instead of 4o C, min instead of Min, h instead of h, either mL or ml, g instead of gram, 10 cm instead of 10cm, µl instead of microlitres etc
5.Page 12, L 13-15, L16-18 correct the sentences
6.Page 17, L12-14, reframe the sentence
7.Page 20, line 8, Replace 'experiments .' with 'experiments.'

Response: We thank this reviewer for the detailed comments and have made the requested changes. All changes have been highlighted in the marked version of the revised manuscript for your convenience.

I found the experiments were executed in a planned way. Technically manuscript is good.

Response: This is very much appreciated.

Reviewer #2: An interesting research work by Lars M Ittner and team.
This is first such study to see interaction profile between human translocator protein and its A147T polymorphism variant.
In summary, the results identified the loss of a subset of interaction partners with the 6 A147T polymorphism variant of human TSPO but one of the positive aspects seems "TSPO's interaction with YWHAQ 7 might contribute to the regulation of TSPO's targeting into mitochondrial membranes involved 8 in apoptotic signaling pathways"
Does author further support it by additional evidence.

Response: We have carefully reworded this sentence to indicate that future investigation is needed to provide direct evidence. It reads now: "Based on DAVID Functional Clustering enrichment analysis, a possible role of the TSPO's interaction with YWHAQ could be the regulation of TSPO's targeting into mitochondrial membranes involved in apoptotic signaling pathways. But future studies are required to provide direct evidence."
